# Extrusion Deformation Mechanism of Mg-8.5Al-1Zn Alloy for Dissolvable Bridge Plugs

**DOI:** 10.3390/ma19081595

**Published:** 2026-04-15

**Authors:** Qinghua Wang, Lifeng Ma, Yanchun Zhu, Liang Ma

**Affiliations:** 1 School of Mechanical Engineering, Taiyuan University of Science and Technology, Taiyuan 030024, China; 2 Heavy Machinery Engineering Research Center of the Ministry of Education, Taiyuan University of Science and Technology, Taiyuan 030024, China; 3 Chongqing Yuhua New Material Technology Co., Ltd., Chongqing 401420, China

**Keywords:** dissolvable magnesium alloy, extrusion deformation, tensile properties, deformation mechanism, EBSD, TEM

## Abstract

To address the problems of coarse grains and unsatisfactory mechanical properties of as-cast Mg-8.5Al-1Zn alloy, which hinder its application in dissolvable bridge plugs, this study took the alloy as the research object and subjected it to plastic deformation via hot extrusion with an extrusion ratio of 12. Through the use of Combined Electron Backscatter Diffraction (EBSD) and Transmission Electron Microscopy (TEM) Testing and Characterization Techniques, the macroscopic mechanical properties, microstructural evolution, and extrusion deformation mechanism of the alloy in both as-cast and as-extruded states were systematically investigated. The results indicate that hot extrusion deformation significantly enhances the comprehensive mechanical properties of the alloy. Compared with the as-cast alloy, the tensile strength, yield strength, and elongation of the as-extruded alloy are increased by 104.0%, 314.9%, and 166.7%, respectively, with the static toughness increasing by 809.1%. The as-cast alloy exhibits coarse grains, Al element segregation, and high-density dislocations. After hot extrusion, dynamic recrystallization dominates the grain refinement, reducing the grain size by approximately 60%. Solute atoms precipitate to form multiphase structures and coherent nano-scale precipitates, along with the formation of tensile twins and a weakened bimodal texture. The improved yield strength of the as-extruded alloy stems from the synergistic effect of multiple strengthening mechanisms, among which precipitation strengthening induced by nano-precipitates is the primary contributor. The enhanced plasticity is attributed to grain refinement and texture regulation. This study clarifies the extrusion deformation mechanism of the Mg-8.5Al-1Zn alloy for dissolvable bridge plugs and verifies the rationality of the hot extrusion process with an extrusion ratio of 12, providing technical support for its industrial application in dissolvable bridge plugs and the performance regulation of similar dissolvable magnesium alloys.

## 1. Introduction

In oil and gas well drilling and completion operations, dissolvable bridge plugs, as key downhole tools, play an important role in temporarily plugging the wellbore and isolating drilling fluid from formation fluids, directly ensuring the safe and efficient conduct of fracturing operations. Compared with traditional bridge plugs, dissolvable bridge plugs do not require additional decomposition procedures after the completion of operations and can be completely dissolved in downhole fluids, which not only significantly reduces operational costs but also remarkably improves operational efficiency and has become a research focus in the field of oil and gas development [1,2,3]. As the core constituent material of dissolvable bridge plugs, the mechanical properties of dissolvable magnesium alloys directly determine the service reliability of bridge plugs; therefore, they must possess excellent high strength and good ductility to withstand the complex downhole pressure, temperature environment and operational loads.

Mg alloys have become the preferred materials in the field of soluble downhole tools due to their excellent solubility, low density, and tunable mechanical properties [4]. Among these, the Mg-8.5Al-1Zn alloy demonstrates significant potential for application in soluble bridge plugs, attributed to its reasonable composition ratio. As the primary alloying element, aluminum (Al) contributes to solid solution strengthening, precipitation strengthening, and improved castability. Zinc (Zn), as a minor alloying element, primarily enhances the solid solution strengthening effect, induces more effective lattice distortion, and improves the aging response characteristics to promote the uniform precipitation of the β phase. Consequently, the micro-galvanic corrosion tendency of magnesium alloys is often regulated by the appropriate addition of zinc. However, as-cast Mg-8.5Al-1Zn alloys commonly exhibit issues such as coarse grains, inhomogeneous microstructure, and inferior mechanical properties, which hinder their ability to meet the service requirements of high-performance soluble bridge plugs and severely limit their industrial application. As an effective plastic processing method for improving the microstructure and mechanical properties of magnesium alloys, extrusion deformation can effectively refine the coarse as-cast structure, reduce grain size, and optimize the distribution of the second phase by controlling an appropriate extrusion ratio. This significantly enhances the comprehensive mechanical properties of the alloy, providing an effective approach to addressing the aforementioned challenges [5,6].

The alloy was subjected to extrusion deformation treatment using a process with an extrusion ratio of 12. The macroscopic tensile properties of the as-cast and extruded alloys were systematically tested. Additionally, EBSD and TEM were employed to deeply investigate the microstructure evolution and extrusion deformation mechanism of the alloy from the as-cast to the extruded state [7]. The regulation mechanism of extrusion deformation on the mechanical properties of the alloy was clarified. This study aims to provide a solid theoretical foundation and technical basis for the industrial application of Mg-8.5Al-1Zn alloy in soluble bridge plugs, while also offering a reference for optimizing the plastic processing techniques of similar soluble magnesium alloys.

## 2. Materials and Methods

### 2.1. Experimental Materials and Extrusion Deformation

The material used in this experiment was a self-developed soluble magnesium alloy. Its chemical composition (mass fraction, %) is as follows: Al 8.5, Zn 1.0, Mn 0.25, Ni 0.20, Cu 0.10, RE 0.10, with the balance being Mg. The experimental raw materials were supplied by Chongqing Yuhua New Material Technology Co., Ltd., Chongqing, China, which also completed the smelting and preliminary processing of the alloy.

**Figure 1:**
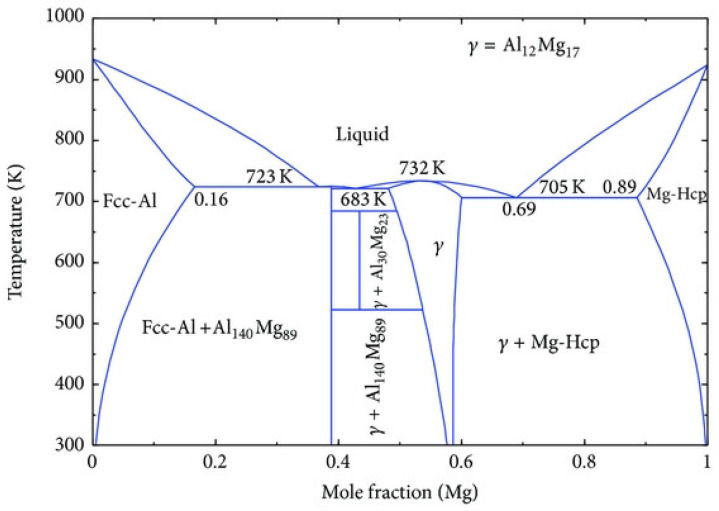
Equilibrium phase diagram of the Mg-Al binary system [8].

The alloy smelting and extrusion process was as follows: First, pure Mg (99.98 wt.%), pure Al (99.98 wt.%), pure Zn (99.5 wt.%), as well as Mn powder, Cu powder, and Ni powder (each with a purity of 99.5 wt.%), along with a small amount of rare earth elements (RE), were added batchwise into a preheated crucible. The crucible was placed in an electric melting furnace and heated until the raw materials were completely melted. Subsequently, primary refining and secondary refining treatments were performed sequentially. After refining, the melt was allowed to stand for a period to ensure compositional homogeneity before casting, ultimately yielding as-cast billets with a diameter of 290 mm and a height of approximately 150 mm. To improve the structural uniformity of the as-cast structure and reduce compositional segregation, a homogenization treatment was performed. From a thermodynamic perspective (Figure 1), the eutectic temperature of the AZ91 alloy in the Mg-Al binary phase diagram is approximately 436 °C [8]. Therefore, a homogenization temperature of 420 °C was selected to ensure an adequate safety margin below the eutectic temperature while maximizing the atomic diffusion rate. From a kinetic standpoint, the homogenization process is governed by the diffusion of aluminum atoms from grain boundaries into the grain interiors. The diffusion coefficient of Al in α-Mg at 420 °C is approximately 10^−14^ m^2^/s. The time required for complete homogenization was calculated to be 15–30 h. Accordingly, a homogenization duration of 24 h was chosen to ensure sufficient elemental homogenization. The homogenized billets were then subjected to extrusion deformation. The extrusion process parameters were set as follows: extrusion temperature of 350 °C, extrusion speed of 3.3 mm/s, and extrusion ratio of 12. The temperature of 350 °C is higher than the recrystallization temperature of the AZ91 alloy, 250–300 °C, but lower than its eutectic temperature, 436 °C, ensuring that dynamic recrystallization (DRX) proceeds completely without the occurrence of incipient melting. The extrusion speed was selected to provide sufficient time for DRX while preventing the formation of adiabatic shear bands. Within the commonly used range for magnesium alloy extrusion, 1–5 mm/s, a speed of 3.3 mm/s was chosen. An extrusion ratio of 12 corresponds to a true strain of approximately 2.48, which is sufficient to achieve full DRX and significant grain refinement. Subsequent experimental results confirmed that this extrusion ratio reduced the average grain size from 17.46 μm to 6.8 μm and significantly enhanced both the strength and ductility of the material. After extrusion, the rods were cooled to room temperature using air cooling and set aside for subsequent use.

### 2.2. Performance Testing

In this study, room-temperature tensile tests were conducted on both the as-cast and extruded alloys using aZwick-Z400E universal testing machine (ZwickRoell, Ulm, Germany) with a maximum load capacity of 250 kN (accuracy class 0.5). The tensile tests were strictly performed in accordance with the GB/T228.1-2021 standard “Metallic Materials—Tensile Testing—Part 1: Method of Test at Room Temperature.” To accurately measure strain, particularly for the determination of proof strength, a clip-on extensometer (ZwickRoell, Ulm, Germany, model: multiXtens) with a gauge length of 35 mm was attached to the specimen. The extensometer had a measuring accuracy of ±0.5% of the reading and remained attached until specimen fracture to capture the full strain response.

Tensile specimens were prepared by cutting along the axial direction from the as-cast ingots and extruded rods, respectively. The specimens were machined into a dog-bone shape conforming to the Type R5 specimen geometry specified in GB/T228.1-2021 [9]. The dimensions were as follows: total length of 64 mm, gauge length of 35 mm, gauge diameter of 5 mm, and a fillet radius of 2.5 mm at the transition between the gauge section and the gripping ends. Both ends featured M10 threads with a length of 12 mm to ensure secure clamping and axial alignment during testing. To minimize the influence of surface defects on the measured properties, the gauge section was mechanically polished to a surface roughness of Ra ≤ 0.4 μm. The tests were conducted at a constant strain rate of 10^−3^ s^−1^ using a closed-loop control system based on the extensometer feedback. Force and strain data were recorded continuously at a sampling rate of 50 Hz. The test was terminated automatically upon specimen fracture, and the force-displacement data were converted to engineering stress-strain curves for subsequent analysis. The following parameters were extracted from the recorded stress-strain data:

Tensile strength (σ_b_, Rm): Defined as the maximum engineering stress sustained during the test, calculated as σ_b_ = Fmax/S_0_, where Fmax is the maximum force and S_0_ is the original cross-sectional area.

Proof strength (σ_0.2_, Rp_0.2_): For magnesium alloys, which typically exhibit a gradual elastic-plastic transition without a distinct yield point, the proof strength was determined using the 0.2% offset method. Specifically, a line parallel to the linear elastic portion of the stress-strain curve was constructed, offset by 0.2% plastic strain. The stress corresponding to the intersection of this offset line with the stress-strain curve was taken as the proof strength (Rp_0.2_). This value represents the stress required to induce 0.2% permanent plastic deformation and serves as the practical yield strength for the material.

Plastic extension (percentage elongation after fracture, A): Calculated as A = (L_u_ − L_0_)/L_0_ × 100%, where L_0_ is the original gauge length (35 mm) and L_u_ is the gauge length measured after fracture. The final gauge length was determined by carefully fitting the fractured halves together and measuring with a digital caliper (accuracy: ±0.01 mm).

Percentage reduction of area (Z): Calculated as Z = (S_0_ − S_u_)/S_0_ × 100%, where S_0_ is the original cross-sectional area and S_u_ is the minimum cross-sectional area at the fracture surface, measured using an optical microscope(ZEISS, Oberkochen, Germany).

For each alloy condition (as-cast and extruded), three independent tensile tests were performed. The reported values for tensile strength, proof strength, elongation, and reduction of area represent the arithmetic mean of the three measurements, accompanied by the standard deviation to indicate the data variability. All statistical calculations were performed using OriginPro 2022 software(OriginLab Corporation, Northampton, MA, USA).

### 2.3. Microstructural Characterization

EBSD analysis was performed using a scanning electron microscope (Zeiss Sigma 300, Carl Zeiss AG, Oberkochen, Germany) equipped with an Oxford Instruments NordlysNano EBSD detector. To obtain high-quality diffraction patterns, the EBSD samples were prepared by means of electrolytic polishing using a solution of 10% nitric acid, 60% methanol, and 30% glycerol (volume ratio) at −20 °C with an applied voltage of 15 V. This electrolyte composition is widely adopted for magnesium alloys to effectively remove surface deformation layers while minimizing surface oxidation [10,11]. The EBSD analysis was conducted with an accelerating voltage of 20 kV and a scanning step size of 0.5 μm. The accelerating voltage of 20 kV was selected to balance pattern quality and spatial resolution, as recommended for magnesium alloys in previous studies [12]. The step size of 0.5 μm was chosen based on the average grain size of the extruded alloy (approximately 8–12 μm), satisfying the requirement that the step size should be less than one-tenth of the grain diameter for statistically reliable orientation measurements [13]. Furthermore, this step size is sufficiently small to resolve {10-12} twin boundaries, which typically have a width of 0.5–2 μm in deformed magnesium alloys [14,15]. For each sample, at least three areas (each 200 × 200 μm^2^) were scanned to ensure that more than 500 grains were analyzed, providing statistically representative data. Orientation Imaging Microscopy (OIM) map acquisition, grain size distribution statistics, and texture analysis were all performed using HKL Channel 5 software (Oxford Instruments, Abingdon, UK). The average grain size was determined from the EBSD orientation maps using HKL Channel 5 software (Oxford Instruments, UK). Grain boundaries were defined as misorientations greater than 15°, which is the commonly accepted threshold for high-angle grain boundaries in magnesium alloys. Twin boundaries with the characteristic {10-12} twin misorientation (86.3° about <11-20>) were excluded from the grain size calculation. For each sample condition, at least three representative EBSD maps (each 200 × 200 μm^2^) were analyzed, covering more than 500 grains to ensure statistical reliability. The equivalent circle diameter (ECD) was calculated for each grain, and the average grain size was obtained as the arithmetic mean of the ECD values.

Transmission electron microscopy (TEM) observations were carried out using a JEM-2100 microscope (JEOL, Akishima, Tokyo, Japan) operated at an accelerating voltage of 200 kV. This accelerating voltage is commonly used for magnesium alloys as it provides sufficient electron beam penetration through the thin regions of the sample while minimizing beam-induced damage [16]. TEM samples were prepared following a standard procedure for magnesium alloys [17]. First, the samples were cut into thin sheets with a thickness of 0.3 mm using a low-speed diamond saw. The sheets were then mechanically ground using silicon carbide abrasive papers down to a thickness of approximately 50 μm. Subsequently, the samples were thinned to electron transparency using a twin-jet electropolishing technique (Struers TenuPol-5). The electrolyte used for twin-jet electropolishing was a mixed solution of 5 vol.% perchloric acid and 95 vol.% ethanol. This electrolyte composition has been widely adopted for magnesium alloys, as the perchloric acid provides effective electrochemical thinning while the ethanol minimizes oxidation during the polishing process [18,19]. The polishing temperature was controlled at −20 °C using a circulating cooling system to suppress excessive chemical reactions and prevent surface oxidation. The polishing voltage was set to 20 V, and the current was monitored to maintain a stable thinning condition. After perforation, the samples were immediately rinsed with ethanol and dried under a warm air stream to preserve the surface quality.

## 3. Results and Discussion

### 3.1. Macroscopic Tensile Properties

**Figure 2:**
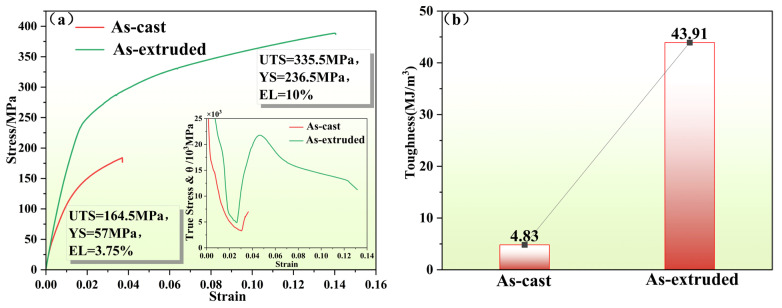
(**a**) Tensile property curve (the inset shows the corresponding strain hardening rate curve); (**b**) static toughness histogram.

Figure 2 presents the work hardening curves and statistical results of tensile properties for the as-cast and extruded states of the Mg alloy. The data in Figure 2b reveal that after extrusion deformation, the static toughness of the as-cast alloy increased from 4.83 MJ/m^3^ to 43.91 MJ/m^3^, representing an increase of 809.1%. Meanwhile, from the work hardening curves in Figure 2a, it can be observed that the overall curve position of the extruded alloy is higher than that of the as-cast alloy. Furthermore, the termination point of work hardening for the extruded alloy corresponds to a higher true stress and greater true strain, demonstrating that the extruded alloy possesses the combined advantages of high strength and good plasticity.

From the tensile curves in Figure 2a, it is evident that the as-cast alloy exhibits a tensile strength of 164.5 MPa, a yield strength of 57 MPa, and an elongation of 3.75%, with all mechanical properties at relatively low levels. After extrusion deformation treatment with an extrusion ratio of 12, the mechanical properties of the alloy were significantly enhanced: the tensile strength reached 335.5 MPa, the yield strength increased to 236.5 MPa, and the elongation increased to 10%. Compared to the as-cast state, the tensile strength, yield strength, and elongation of the extruded alloy improved by 104.0%, 314.9%, and 166.7%, respectively.

The substantial improvement in the mechanical properties of the alloy is primarily attributed to the grain refinement effect, the formation of a favorable texture, and the uniform distribution of the second phase during the extrusion deformation process [20,21,22]. The mechanical properties of the alloy after extrusion meet the service requirements for soluble bridge plugs (tensile strength ≥ 300 MPa, yield strength ≥ 220 MPa), indicating that an extrusion ratio of 12 is suitable for the plastic deformation processing of this alloy and effectively optimizes its mechanical properties. This provides performance support for the application of this alloy in soluble bridge plugs.

### 3.2. Microstructural Characteristics

#### 3.2.1. X-Ray Diffraction Analysis

**Figure 3:**
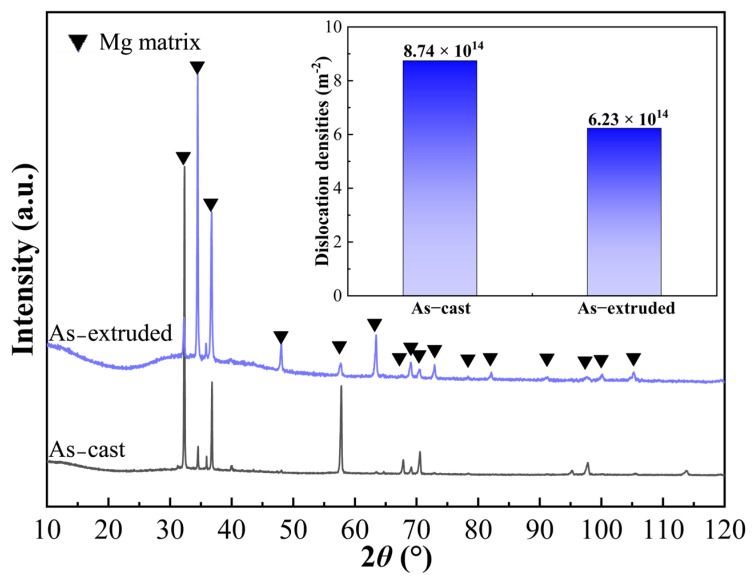
XRD spectra of as-cast and extruded Mg alloys and dislocation density calculated based on XRD analysis.

Figure 3 presents the microstructural characterization results of the as-cast alloy. In the X-ray Diffraction (XRD) pattern, only distinct peaks corresponding to the Mg matrix were observed, while no significant diffraction peaks from secondary phases were detected. This may be attributed to the relatively high solid solubility of the alloying elements during the casting cooling process, or to the volume fraction of precipitates in the as-cast state being too low, falling below the detection limit of X-ray diffraction. Furthermore, based on calculations from full-pattern fitting of the XRD data, the dislocation density of the as-cast alloy was determined to be as high as 8.74 × 10^14^ m^−2^. This indicates the presence of significant lattice distortion within the as-cast structure, reflecting relatively high internal stresses in the as-cast alloy.

#### 3.2.2. Grain Structure and Orientation Analysis

**Figure 4:**
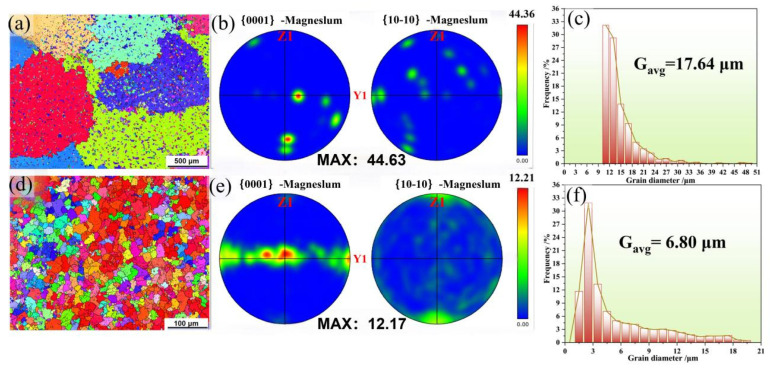
Grain boundary (GB) map, interplanar grain fraction (IPF) map, and average grain size map during the casting and transformation states: (**a**–**c**) As-cast; (**d**–**f**) As-deformed.

Figure 4 presents the grain boundary (GB) maps, inverse pole figure (IPF) maps, and average grain size statistical results for the as-cast and extruded states of the soluble magnesium alloy. The core performance requirements for magnesium alloys used in soluble bridge plugs are “high strength, high plasticity, and controllable dissolution.” The quality of microstructural reconstruction during the extrusion process directly determines the effectiveness of alloy property optimization. Regarding the synergistic enhancement of strength and plasticity, the grain refinement effect resulting from the reduction in grain size from 17.64 μm to 6.8 μm and the dispersion strengthening from secondary phase precipitation collectively contribute to a significant increase in both yield strength and tensile strength of the alloy [23,24]. Meanwhile, texture weakening, the formation of a bimodal texture, and the activation of multiple slip systems effectively improve the plasticity and deformation uniformity of the alloy, avoiding the brittle fracture issues prone to occur in the as-cast alloy. This achieves a synergistic optimization of strength and plasticity, enabling the alloy to meet the service requirements of bearing pressure and resisting deformation for downhole bridge plugs. Among these mechanisms, grain refinement dominated by dynamic recrystallization is the core mechanism for enhancing the strength and plasticity of the alloy, while texture regulation and dislocation balance further optimize the strength–plasticity matching relationship [25].

As shown in Figure 4a–c, the as-cast alloy exhibits coarse grain characteristics with an average grain size of 17.46 ± 2.3 μm. The grain orientation displays a random distribution without any obvious preferred texture, as reflected in the corresponding inverse pole figure (IPF) map and pole figures. This phenomenon arises because the as-cast alloy forms through direct solidification, where grains grow freely without external constraints, resulting in a uniformly distributed orientation without significant preference. Figure 4d–f reveal that after extrusion deformation, the coarse-grain structure of the as-cast alloy is completely disrupted, and the grains are significantly refined, forming a uniform and dense equiaxed grain structure. At this stage, the average grain size of the alloy is reduced to 6.8 ± 0.9 μm, representing an 61.1% reduction compared to the as-cast alloy. This remarkable grain refinement is a direct manifestation of dynamic recrystallization (DRX) during the extrusion process, where the stored deformation energy drives the nucleation and growth of new, strain-free grains [26,27,28]. The refined grain structure serves as the core foundation for the enhanced strength and plasticity of the alloy, consistent with the Hall–Petch relationship.

Simultaneously, texture, as a key factor influencing the mechanical properties and anisotropy of magnesium alloys, has its intensity and morphological changes directly determining the plasticity level and deformation uniformity of the alloy [29,30]. This occurs because, although the overall orientation shows no significant preference due to free grain growth during casting, localized strong orientation aggregation forms under the influence of compositional segregation and coarse grains. After hot extrusion treatment, the texture intensity of the alloy is significantly weakened, with MAX decreasing to 12.17, and a typical bimodal texture characteristic forms [31,32,33]. This bimodal texture consists of two grain components with different orientations: one component comprises grains with preferred orientations close to the basal plane, while the other consists of grains with preferred orientations deviating from the basal plane. These two grain orientations complement each other, forming a uniform orientation distribution state [34,35]. The weakening of the texture and the formation of the bimodal structure effectively reduce the anisotropy of the alloy, providing an important guarantee for the improvement of alloy plasticity.

### 3.3. Analysis of Extrusion Deformation Mechanism

#### 3.3.1. Microstructural Characteristics and Solidification Behavior Analysis of the As-Cast Alloy

**Figure 5:**
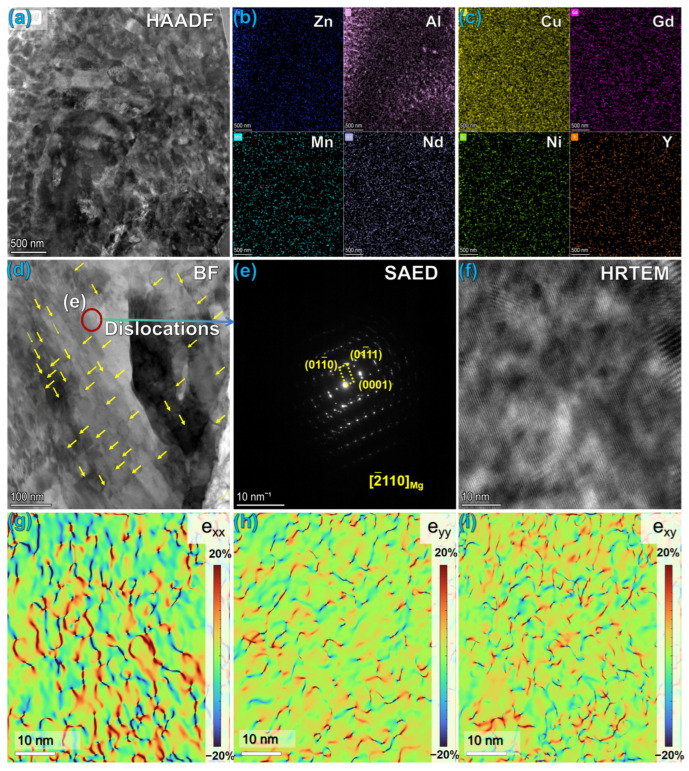
Microstructural characterization of as-cast Mg alloy: (**a**) Low-magnification HAADF images; (**b**,**c**) Corresponding EDS surface scan distributions of Al and other elements; (**d**) Bright-field images revealing short and dense dislocation features in the matrix; (**e**) Selective area electron diffraction (SAED) patterns; (**f**) High-resolution (HRTEM) images of the matrix; (**g**–**i**) Corresponding to (**f**) and geometric phase analysis (GPA) strain field distribution maps based on the XX (0001) and YY (01-10) crystal planes.

Combined with the TEM analysis results in Figure 4, the origin mechanism of microstructural defects in the as-cast alloy can be further revealed. The High-Angle Annular Dark-Field (HAADF) image (Figure 5a) and its corresponding Energy-Dispersive Spectroscopy (EDS) mapping (Figure 5b,c) show that the Al element exhibits significant segregation characteristics along grain boundaries and interdendritic regions, while other alloying elements are relatively uniformly distributed in the matrix [36]. According to solidification theory, this phenomenon represents typical non-equilibrium solidification characteristics. Due to the equilibrium partition coefficient of Al in the Mg matrix being k < 1, during the solidification process of the alloy, solute atoms (Al) are continuously rejected into the liquid phase, ultimately resulting in solute enrichment in the last-solidified grain boundaries and interdendritic regions. This inhomogeneity in chemical composition induces local lattice mismatch, thereby triggering the formation of microstructural defects.

The bright-field image (Figure 5d) further validates the XRD calculation results shown in Figure 3, where a large number of short and densely distributed dislocation lines can be observed within the Mg matrix. These dislocation morphology characteristics are typically closely related to the thermal stress and constitutional supercooling generated during the casting solidification process. Due to the difference in thermal expansion coefficients between the solute-enriched grain boundaries and the matrix, thermal mismatch stress develops during cooling, which serves as a driving force inducing the nucleation and multiplication of dislocations. The high-resolution TEM (HRTEM) image (Figure 5f) and its corresponding geometric phase analysis (GPA, Figure 5g–i) intuitively present the stress state within the as-cast alloy. GPA strain field analysis was conducted on the XX direction (0001) and YY direction (01−11) crystal planes of the Mg matrix, revealing the distribution of high-density lattice distortion regions within the matrix. These atomic-scale microstructural defects not only account for the broadening of XRD peaks but also confirm that the as-cast alloy stores relatively high distortion energy due to stress accumulation during the solidification process. This distortion energy provides sufficient driving force for microstructural evolution (such as dynamic recrystallization) during subsequent thermal processing [37].

#### 3.3.2. Microstructural Evolution and Dynamic Recrystallization of the Extruded Alloy

**Figure 6:**
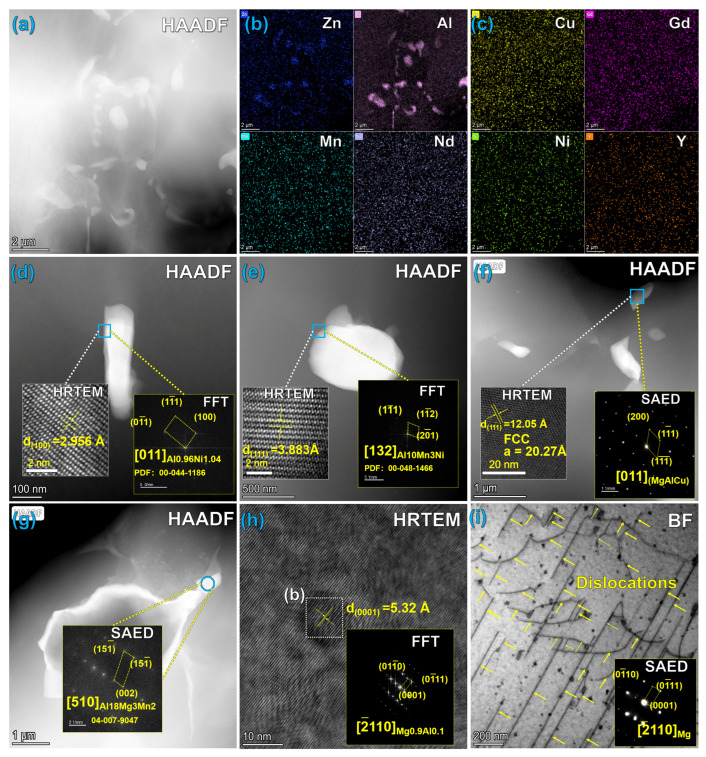
Microstructure and phase analysis of compressed Mg alloy: (**a**) Low-magnification HAADF images; (**b**,**c**) Elemental EDS surface scan distributions; (**d**–**h**) Morphologies, high-resolution images, and SAED patterns of different precipitates (AlNi, AlMnNi, AlMgCu, etc.); (**i**) Long straight dislocation morphologies in the compressed matrix and SAED patterns along the [−2110] crystal axis.

After hot extrusion deformation with an extrusion ratio of 12, the microstructure of the alloy underwent dramatic reconstruction, with specific characteristics shown in Figure 6.

##### Precipitation Behavior of Precipitates

Compared with the as-cast microstructure, the most significant feature of the extruded alloy microstructure is the precipitation of solute atoms, which alters the segregation state observed in the as-cast condition. EDS mapping (Figure 6b,c) reveals that Al and Zn elements no longer exhibit the grain boundary and interdendritic segregation characteristics seen in the as-cast state; instead, they extensively precipitate, forming secondary phases with coexisting micron and nanometer scales. Combined with selected area electron diffraction (SAED) and fast Fourier transform (FFT) analysis results, it is confirmed that the extruded alloy contains a complex multiphase structure, specifically including Al_0.96_Ni_1.04_, Al_10_Mn_3_Ni, Al_18_Mg_3_Mn_2_ phases, as well as an AlMgCu phase with a face-centered cubic (FCC) structure (lattice constant a = 2.027 nm).

**Figure 7:**
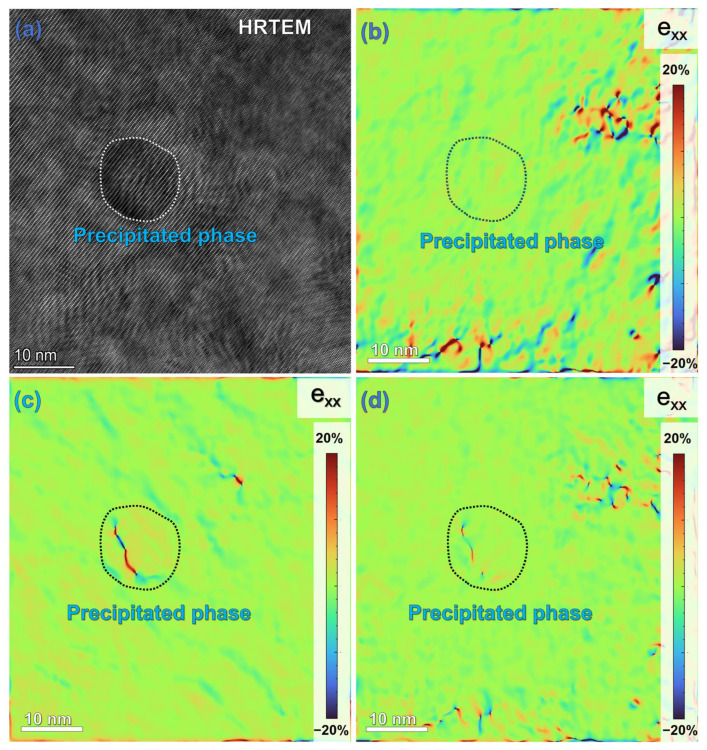
GPA distribution at the interface between nanoscale Mg_0.9_Al_0.1_ precipitates and the matrix in the compressed alloy: (**a**) High-resolution image of the precipitates; (**b**–**d**) GPA distribution based on the Mg matrix’s XX (0001) and YY (01-10) crystal planes.

Particularly noteworthy is the observation in Figure 6h of Mg_0.9_Al_0.1_ nanoprecipitates with a size of approximately 15 nm. GPA analysis results (Figure 7b–d) indicate significant strain concentration within these Mg_0.9_Al_0.1_ nanophases, while the strain concentration outside the phase interface with the matrix is relatively low. This characteristic suggests that these nanoprecipitates maintain a good coherent relationship with the Mg matrix [38]. During the hot extrusion process, severe plastic deformation introduces a large number of lattice defects within the alloy, providing abundant nucleation sites for the precipitation of solute atoms. These uniformly dispersed Mg_0.9_Al_0.1_ nanophases are one of the key factors contributing to the significant improvement in the mechanical properties of the extruded alloy [39,40].

##### Dislocation Evolution and Dynamic Recrystallization

XRD calculation results (Figure 3) show that the dislocation density of the extruded alloy is significantly reduced compared to the as-cast state, decreasing to 6.23 × 10^14^ m^−2^. The TEM bright-field image (Figure 6i) further reveals a significant transformation in the dislocation morphology of the alloy: from the “short and densely distributed” characteristic in the as-cast state to a “long, straight, and sparsely distributed” morphology in the extruded state. This morphological change is a typical feature and direct evidence of the occurrence of dynamic recrystallization and dynamic recovery during the hot extrusion process.

During the hot extrusion process, significant dynamic recovery occurs in the Mg matrix, where dislocations undergo rearrangement through movements such as climb and cross-slip, with dislocations of opposite signs annihilating each other, effectively reducing the dislocation density. Simultaneously, new recrystallized grains nucleate and grow at grain boundaries or in regions with high dislocation density, consuming the high distortion energy stored in the as-cast structure and further contributing to the decrease in dislocation density. The appearance of long, straight dislocations in the extruded alloy indicates that the obstacles to dislocation motion on specific slip systems (such as basal slip) are relatively uniform, and some dislocations are pinned by uniformly dispersed nanoprecipitates, thus forming the characteristic long, straight, and sparse dislocation morphology.

##### Twinning Behavior and Stacking Faults

**Figure 8:**
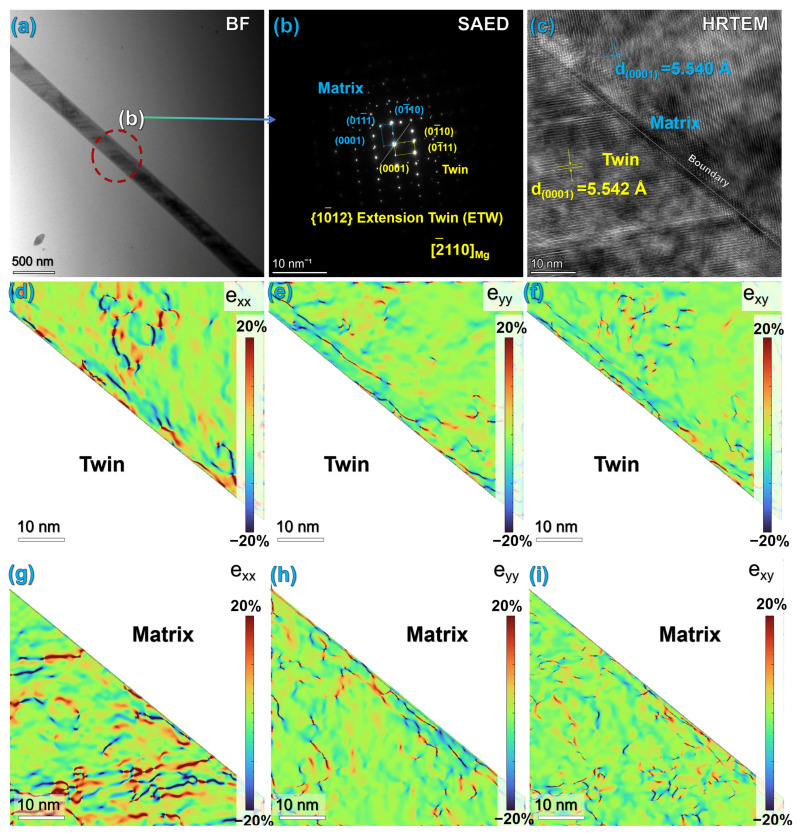
Analysis of Deformed Twin and Interface Structure in Compressed Mg Alloy: (**a**) Bright-field image of twin morphology; (**b**) SAED pattern calibrated as {10-12} tensile twin; (**c**) High-resolution image of internal dislocation in the twin; (**d**–**i**) Analysis of GPA strain field at the twin boundary and within the twin.

In addition to slip, twinning is also an important plastic deformation mode for magnesium alloys and plays a key role in coordinating plastic deformation during the extrusion process. Figure 8a shows the presence of obvious deformation twins in the microstructure of the extruded alloy. Through selected area electron diffraction (SAED) calibration, these twins were identified as {10-12} tensile twins. This type of tensile twin is typically activated under stress conditions involving tension along the c-axis or compression perpendicular to the c-axis, effectively coordinating plastic deformation during the extrusion process, relieving local stress concentration, and improving the deformation uniformity of the alloy [41,42,43].

High-resolution TEM images (Figure 8c) reveal a distinct stacking fault structure within the twins. This phenomenon arises from the low stacking fault energy of magnesium alloys. During plastic deformation, the formation and expansion of stacking faults induce twin nucleation and growth, and this stacking fault structure represents the typical trace left after stacking fault-induced twin formation.

Strain field analysis of the matrix/twin interface using GPA technology (Figure 8d–i) revealed significant strain concentration zones at the interface between the matrix and the twin [44]. This indicates that the twin boundary, as a high-energy interface, effectively impedes dislocation motion, causing dislocations to accumulate at the interface and thereby generating stress concentration. Additionally, noticeable stress concentration can be observed within the twin interior, particularly along the (0001) crystal plane direction, a phenomenon directly related to the presence of stacking faults inside the twin. In summary, the stacking faults within the twins and the twin boundaries together constitute a dual barrier that hinders dislocation slip, effectively enhancing the mechanical properties of the alloy and playing an important role in the strengthening of the extruded alloy [45].

### 3.4. Discussion on Strengthening and Toughening Mechanisms

From the comparison of room-temperature tensile properties between the as-cast and extruded alloys shown in Figure 2, it can be observed that after hot extrusion deformation treatment, the comprehensive mechanical properties of the alloy achieved a leapfrog improvement. The material’s ability to absorb energy before fracture was significantly enhanced. The specific strengthening and toughening mechanisms are analyzed as follows.

#### 3.4.1. Strengthening Mechanisms of Yield Strength

Although the dislocation density of the extruded alloy (6.23 × 10^14^ m^−2^) is lower than that of the as-cast alloy (8.74 × 10^14^ m^−2^), indicating that the contribution of dislocation strengthening to the yield strength of the alloy has actually diminished, the yield strength of the extruded alloy still increased by nearly four times compared to the as-cast state. Combined with the previous microstructural analysis results, it can be concluded that this significant improvement is primarily attributed to the synergistic effects of the following strengthening mechanisms:

(a) Precipitation Strengthening: This is the most important strengthening source for the improvement in yield strength of the extruded alloy. TEM observation results reveal the presence of a large number of uniformly dispersed nanoscale precipitate particles in the extruded alloy. During dislocation motion, dislocations need to bend to bypass these precipitates, leaving behind dislocation loops, a process that significantly increases the critical resolved shear stress for dislocation movement. Meanwhile, GPA analysis shows significant strain concentration within the precipitates, a characteristic confirming that these nanoscale precipitates can act as strong obstacles, effectively pinning dislocation motion and further enhancing the yield strength of the alloy.

(b) Grain Refinement Strengthening: According to the grain size data from EBSD measurements in Figure 4, the dynamic recrystallization occurring during the extrusion process leads to significant grain refinement. Based on the Hall–Petch relationship, grain refinement results in a substantial increase in grain boundary density. As natural barriers to dislocation slip, grain boundaries effectively impede the transmission of dislocation slip, thereby significantly enhancing the yield strength of the alloy.

(c) Twinning and Interface Strengthening: GPA analysis results confirm the presence of high-density strain concentration at twin boundaries and matrix/twin interfaces. Twin boundaries partition the original grains, producing a dynamic Hall–Petch effect that further reduces the mean free path of dislocations, hinders dislocation slip, and consequently achieves an improvement in the yield strength of the alloy.

(d) Texture Regulation Effect: The weakening of texture intensity and the formation of a bimodal texture in the extruded alloy effectively reduce the anisotropy of the alloy, improve the uniformity of dislocation motion, provide an important guarantee for the improvement of alloy plasticity, and indirectly synergistically enhance both the yield strength and the strength–plasticity matching of the alloy.

#### 3.4.2. Work Hardening Behavior and Plasticity Improvement

The significant increase in elongation (from 3.75% to 10%) is primarily attributed to the replacement of the coarse as-cast dendritic structure by the dynamically recrystallized microstructure, which eliminates solidification defects and stress concentration points present in the as-cast state. Additionally, the activation of {10-12} tensile twins provides additional deformation degrees of freedom, accommodates strain along the c-axis direction, and delays crack initiation.

The work hardening curves in Figure 2a show that the extruded alloy sample exhibits the highest work hardening rate, a characteristic closely related to the distribution characteristics of nanoprecipitates observed by means of TEM. In the early stage of tensile deformation, dislocations undergo rapid multiplication, and these multiplied dislocations are effectively intercepted by the high-density, uniformly dispersed nanoprecipitates and twin boundaries, leading to dislocation accumulation and the rapid establishment of back stress. This effectively maintains the alloy’s high work hardening capacity, delays the occurrence of necking during the tensile process, and provides a guarantee for the continuous progress of plastic deformation in the alloy.

The elongation of the extruded alloy significantly increased from 3.75% in the as-cast state to 10%. This plasticity improvement mainly benefits from the synergistic effects of microstructural optimization and deformation mechanisms. On one hand, the uniform and fine equiaxed grain structure formed by dynamic recrystallization replaces the coarse dendritic structure of the as-cast state, effectively eliminating solidification defects (such as compositional segregation and lattice distortion) and stress concentration points present in the as-cast alloy, reducing the sources of crack initiation during deformation. On the other hand, the activation of {10-12} tensile twins provides additional deformation degrees of freedom for plastic deformation of the alloy, effectively accommodating strain along the c-axis direction, relieving local stress concentration, and delaying crack initiation and propagation, thereby significantly enhancing the elongation and plastic deformation capacity of the alloy.

## 4. Conclusions

This paper investigated the Mg-8.5Al-1Zn alloy for soluble bridge plugs, subjecting it to plastic deformation treatment using a hot extrusion process with an extrusion ratio of 12. Through systematic macroscopic performance testing and microstructural characterization, the extrusion deformation mechanism and strengthening-toughening mechanisms of the alloy were deeply explored, leading to the following main conclusions:

1. Hot extrusion deformation significantly optimized the macroscopic mechanical properties of the Mg-8.5Al-1Zn alloy. The tensile strength, yield strength, and elongation of the extruded alloy reached 335.5 MPa, 236.5 MPa, and 10%, respectively, representing increases of 104.0%, 314.9%, and 166.7% compared to the as-cast state, with static toughness increasing by 809.1%, confirming that an extrusion ratio of 12 is an appropriate plastic processing parameter for this alloy. Simultaneously, soluble magnesium alloy bridge plug materials are typically required to meet the following specifications [4,46,47]: σ_b_ ≥ 310 MPa, σ_0.2_ ≥ 220 MPa, and A ≥ 6%. These parameters indicate that the alloy under such conditions satisfies the required performance criteria.
2. The as-cast Mg-8.5Al-1Zn alloy exhibited coarse randomly oriented grains (average size 17.46 μm), with Al element segregation along grain boundaries and interdendritic regions, and contained high-density dislocations (8.74 × 10^14^ m^−2^) and significant lattice distortion. These characteristics originated from solute rejection, thermal mismatch stress, and constitutional supercooling during non-equilibrium solidification, providing sufficient distortion energy for microstructural reconstruction during subsequent extrusion deformation.
3. During hot extrusion, the alloy underwent dramatic microstructural reconstruction: dynamic recrystallization dominated grain refinement, reducing the average grain size to 6.8 μm and forming a uniform and dense equiaxed grain structure. The solute segregation present in the as-cast state disappeared, with elements such as Al and Zn precipitating out to form multiphase structures including Al_0.96_Ni_1.04_ and Al_10_Mn_3_Ni, as well as Mg_0.9_Al_0.1_ nanoprecipitates approximately 15 nm in size that maintained coherency with the matrix. Dislocation morphology transformed from “short and densely distributed” to “long, straight, and sparsely distributed,” with dislocation density decreasing to 6.23 × 10^14^ m^−2^. Simultaneously, {10-12} tensile twins and a weakened bimodal texture formed (texture intensity decreasing from 44.63 to 12.17).
4. The significant improvement in the yield strength of the extruded alloy resulted from the synergistic effects of precipitation strengthening, grain refinement strengthening, and twinning/interface strengthening, with precipitation strengthening from uniformly dispersed nanoprecipitates being the primary contributor. The increase in work hardening rate was related to nanoprecipitates and twin boundaries intercepting dislocations and establishing back stress, while plasticity improvement benefited from grain refinement through dynamic recrystallization, {10-12} tensile twins providing additional deformation degrees of freedom, and the bimodal texture reducing anisotropy of the alloy.
5. This study clarified the extrusion deformation mechanism and strengthening-toughening mechanisms of the Mg-8.5Al-1Zn alloy and verified the optimization effect of the hot extrusion process with an extrusion ratio of 12 on the alloy’s properties, providing solid theoretical support and a technical basis for the industrial application of this alloy in soluble bridge plugs. It also offers a reference for the plastic processing optimization and performance control of similar soluble magnesium alloys.

## Data Availability

The original contributions presented in this study are included in the article. Further inquiries can be directed to the corresponding author.
